# Primitive Neuroectodermal Tumor with Kidney Involvement: A Case Report

**DOI:** 10.5812/iranjradiol.4661

**Published:** 2014-05-15

**Authors:** Davood Sharifi Doloui, Tahereh Fakharian, Vahid Yahyavi, Sirous Nekooei, Hamid Reza Zivarifar, Kamran Ghafarzadegan

**Affiliations:** 1 Department of Gastroenterology and Liver Disease, Ghaem Hospital, Mashhad University of Medical Sciences, Mashhad, Iran; 2 Department of Radiology, Ghaem hospital, Mashhad University of Medical Sciences, Mashhad, Iran; 3 Department of Pathology, Razavi hospital, Mashhad, Iran

**Keywords:** Neuroectodermal Tumors, Primitive, Neoplasm, Abdomen, Kidney, Neoplasm Metastasis, Stomach

## Abstract

Primitive neuroectodermal tumor (PNET) is usually an aggressive, rapidly progressing and metastasizing tumor. Occurrence of this type of tumor in the kidney is considered as unusual and few cases have been reported so far. We present a metastatic PNET arising probably from the kidney in a 17-year-old female patient with local invasion and metastasis to the stomach. PNET should be considered as a differential diagnosis of a large heterogeneous soft tissue mass in the abdomen, especially in those with widely local invasion and metastases.

## 1. Introduction

Primitive neuroectodermal tumors (PNET) are a group of small round cell malignancies with neural crest origin(1). These tumors were first described by Su (2). They are highly aggressive and rare. These tumors may originate in the central nervous system or in other tissues peripherally. The peripheral type is seen typically in the soft tissues of the chest wall and paraspinal region and unusually along the genitourinary tract. PNET rarely presents as an organ-derived neoplasm (3). Peripheral PNETs arising in the abdomen are rare (1). The overall incidence of peripheral PNET is 1% of all sarcomas (with the incidence of sarcoma about 30/million (4) or less than 1% of all malignant tumors). The incidence of PNET in the abdomen and pelvis, including the retroperitoneum, is about 14% of all peripheral PNETs (5). Renal PNET is extremely rare with fewer than 50 cases in the literature (6). We report here a case of retroperitoneal PNET, most probably of renal origin, with metastasis to the stomach as an unusual case.

## 2. Case Presentation

A 17-year-old girl was admitted with abdominal pain and distension for the past 6 months. She also had nausea, vomiting and a weight loss of about 10 kg. Physical examination revealed multiple abdominal masses on the left side of the abdomen, pallor, ascites and pleural effusion without any peripheral lymphadenopathy. The patient’s remarkable laboratory findings were severe anemia (hemoglobin: 7.4 g/dL) and a significantly increased ESR (85 mm/h) and serum LDH (835 U/L).

**Figure 1.**
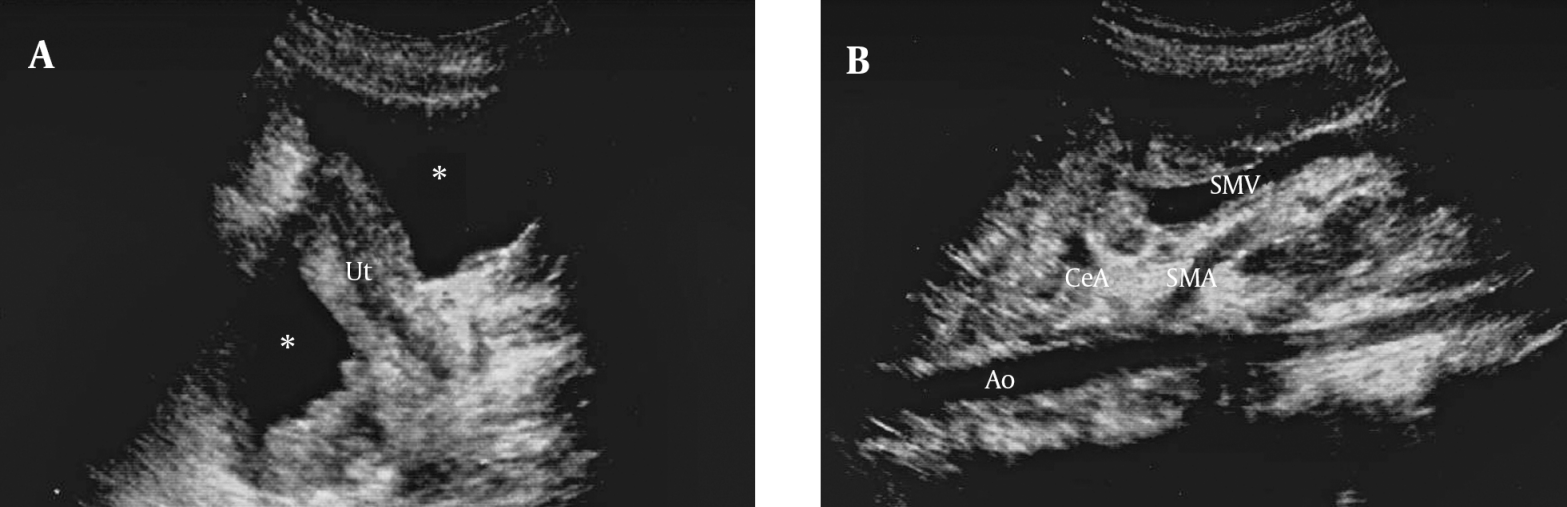
Abdominopelvic ultrasonography of the patient, A) Free fluid (*) in the pelvic cavity, Ut= uterus; B) Encasement of the celiac artery (CeA) and superior mesenteric artery (SMA) by mass lesion, Ao=aorta, SMV= superior mesenteric vein.

**Figure 2.**
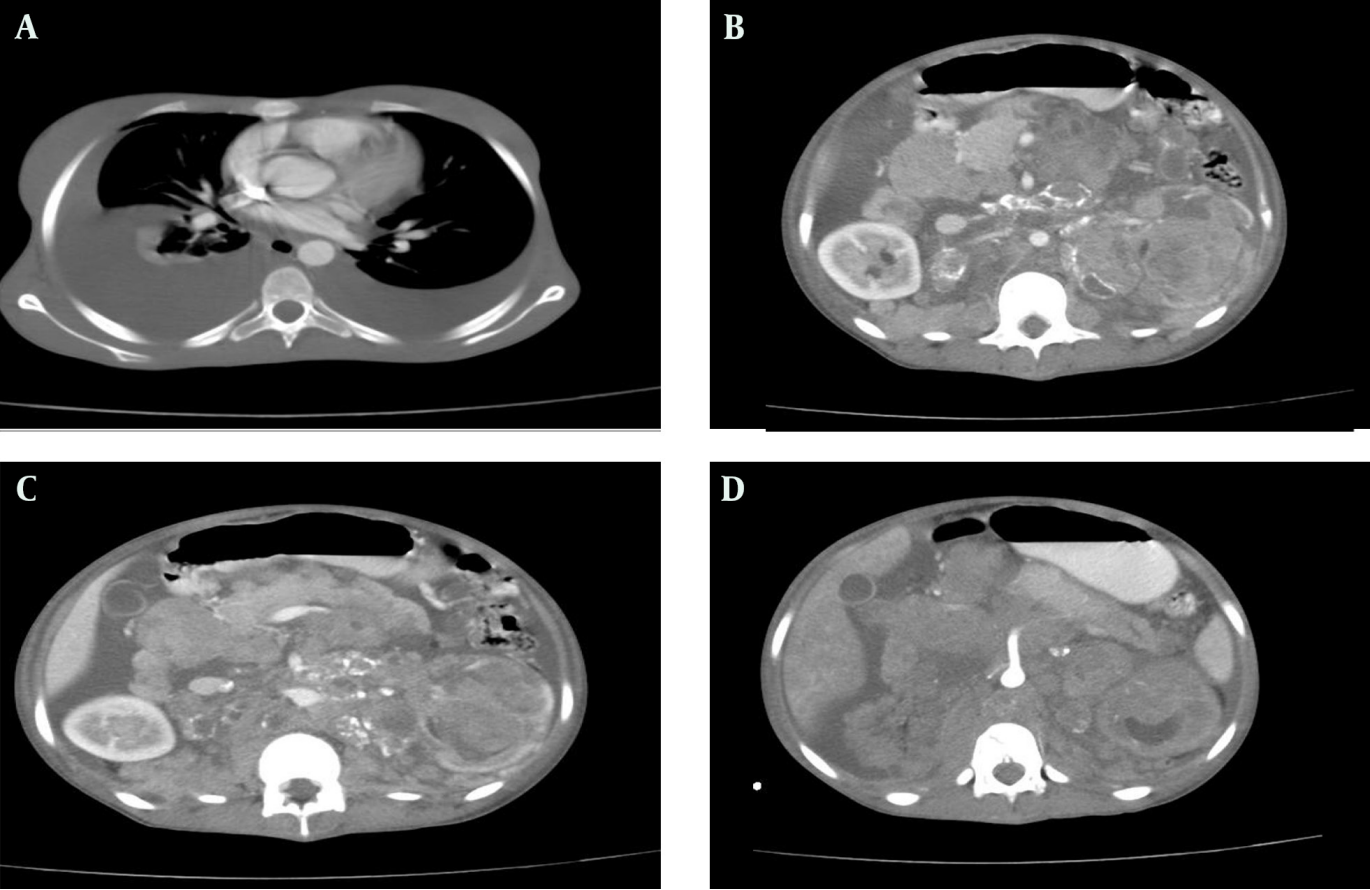
Thoracoabdominal CT scan of the patient with IV and oral contrast, A) Bilateral pleural effusion; B) Left kidney mass with heterogeneous enhancement; C) The pancreas is pushed anteriorly; D) Encasement of the superior mesenteric artery by mass lesion

Abdominal ultrasonography revealed mass lesions adherent to each other in the upper portion of the retroperitoneal space. There was free fluid in the peritoneal and both pleural cavities (Figures 1 and 2). Paracentesis was performed and the ascitic fluid and pleural effusion were bloody. Analysis of both fluids showed exudative features with negative cytopathology.

**Figure 3.**
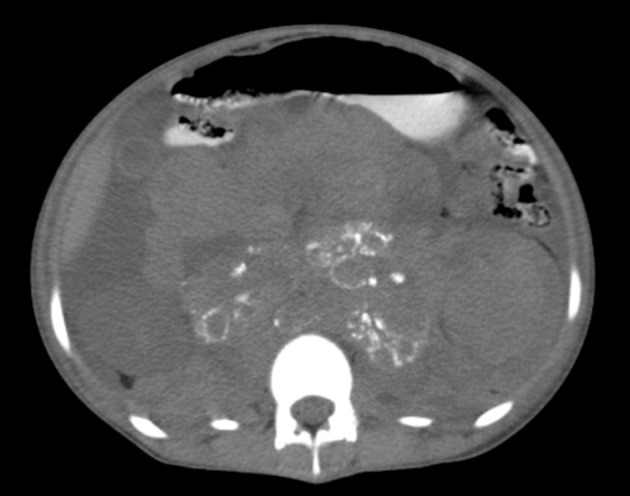
Abdominal CT scan of the patient with oral without IV contrast shows calcification of the tumoral mass

Abdominal CT scan confirmed several adherent mass lesions in the retroperitoneum with heterogeneous solid and cystic components (mostly solid) and several curvilinear calcifications (Figure 3). The left kidney was destructed with no obvious contrast secretion (Figure 2). The pancreas was displaced anteriorly (Figure 2). The abdominal aorta, the inferior vena cava and the visceral vessels were encased by the mass lesions (Figures 1 and 2). Moderate hydronephrosis of the right kidney was evident. The liver, spleen, uterus and both ovaries were intact. There was no evidence of metastasis to the stomach in CT scan. Malignant retroperitoneal tumor (probably originating from the left kidney) such as sarcoma, neuroblastoma, or other malignant tumors were suggested as differential diagnoses. The interventional radiologist planned a percutaneous needle biopsy for definitive diagnosis. A 75×70 mm, mass lesion was localized in the left kidney by ultrasound at the posterior axillary line at T11-T12 intercostal space. Under local anesthesia, two biopsy specimens were obtained by a 16G core biopsy needle.

**Figure 4.**
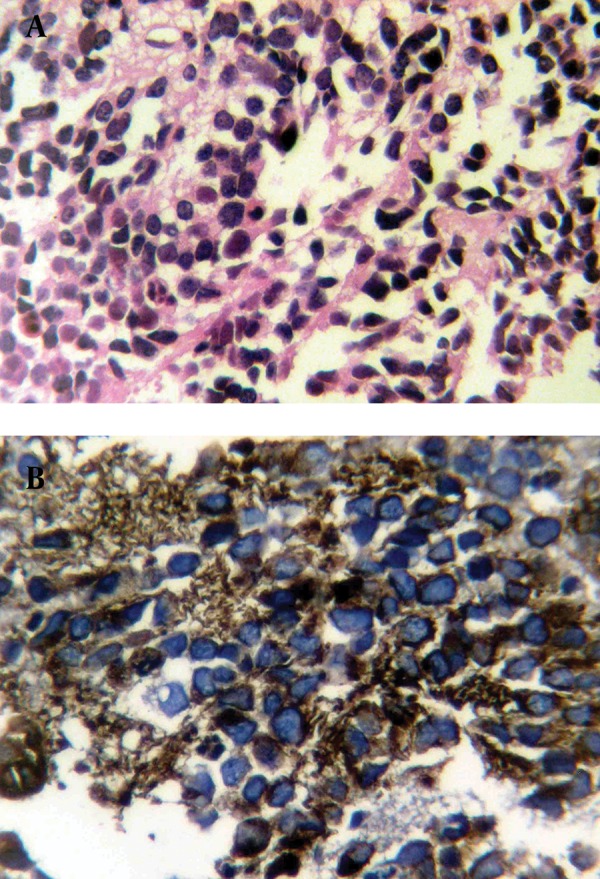
Histopathology of PNET tumoral cells, A) Hematoxylin and eosin staining, 100×; B) Immunohistochemical staining positive for CD99

Histological examination revealed a small rounded cell tumor with hyperchromatic nucleus and scant cytoplasm in a fibrotic stroma. Foci of necrosis were remarkable (Figure 4). Immunohistochemical finding was compatible with PNET or Ewing’s sarcoma (positive for neuron specific enolase (NSE), vimentin, CD99 and negative for desmin, leukocyte common antigen (LCA) and chromogranin) (Figure 4). Biopsy of a 5mm nodule in the cardia of the stomach that was discovered during an upper endoscopy for intractable nausea and vomiting showed the same pathology. As a result of local invasion and metastasis, the tumor was unresectable and the patient was referred to an oncologist for chemotherapy. Now, after 6 months, the patient is on multi-drug chemotherapy. The tumor has shown regression in follow-up ultrasonography, but is still unresectable.

## 3. Discussion

Peripheral primitive neuroectodermal tumors (peripheral PNETs) are a group of small round cell malignancies with neural crest origin that arise outside the central and sympathetic nervous system (1). They belong to the Ewing sarcoma family of tumors (EFT), because of their similar histologic and immunohistochemical characteristics (7). Both PNETs and Ewing sarcomas strongly express the glycoprotein p30/32 (CD99), which is encoded by the microneme protein 2 (MIC2) genes (8).

Peripheral PNETs are rare in the abdomen (1). This tumor rarely presents as an organ-derived neoplasm (3). In recent years, there have been several reports of visceral PNETs arising in different organs, including the lung, pancreas, jejunum, common hepatic duct, kidney and uterus (1). Renal localization is very rare. PNET tumors of the kidney usually affect young individuals, are large at presentation and tend to be highly aggressive tumors (9). There are almost 50 cases reported in the literature, although it is difficult to estimate the exact number since often it has not been differentiated from Ewing’s sarcoma (10). Carefully selected immunohistochemical panel is important for differentiating this tumor from other small round cell tumors of the kidney such as rhabdomyosarcoma, neuroblastoma, clear cell sarcoma of the kidney, desmoplastic small round cell tumor (DSRCT), carcinoid tumor, nephroblastoma and Ewing’s sarcoma. Immunohistochemically, PNET cells express vimentin, NSE and CD99 (9). The positive reactivity to CD99 is a clue for PNET diagnosis (3). Effective local and systemic therapies are necessary for the treatment of PNET. Most chemotherapy regimens are a combination of cyclophosphamide, doxorubicin (adriamycin), vincristine, dactinomycin, ifosphamide and etoposide. Both surgery and radiation therapy are effective local therapies for the primary lesion (11).

Review of radiologic findings showed that most cases of retroperitoneal PNETs in the literature, similar to our case, presented as a large mass with areas of necrosis and heterogeneous enhancement in contrast imaging. Some cases had areas of hemorrhage, but in our case, there was no hemorrhage. Our case had several foci of curvilinear calcification compared to other case reports, in which calcification was a rare finding (one of ten cases in a case series) (5). There were septation and intratumoral cystic components similar to our case in some of the PNETs arising from the kidney in the literature, so it could mimic cystic renal cell carcinoma (5). Organ invasion and displacement, such as direct invasion or displacement of the pancreas, kidney, spleen and stomach was present in some cases, including our case (5). There were no cases of retroperitoneal PNET presenting with ascites and pleural effusion in the literature.

Although vascular encasement of abdominal aorta and visceral vessels was seen in our case, like some other cases of retroperitoneal PNETs, there was no clinical or imaging evidence of venous thrombosis in the inferior vena cava (IVC), while venous thrombosis has been detected in other patients (5, 9). Approximately 20% of the patients present with metastatic disease; out of which 44% present with lung metastases only,51% have bone or bone marrow involvement (with or without lung metastases) and less than 5% present with metastases in other organs (11), like our case with metastasis to the stomach. Imaging manifestations of peripheral PNETs include large soft tissue masses with cystic and necrotic areas and heterogeneous enhancement after intravenous administration of contrast materials (11). Fine tumor calcification is rarely seen (12). Although it is difficult to distinguish them from other types of soft tissue sarcomas only based on imaging, the possibility of peripheral PNETs should be considered when a large heterogeneous soft tissue mass is detected in the abdomen, especially in those with widely local invasion and metastases and in young adults with a large kidney mass (1).
